# Associations of physical activity participation trajectories with subsequent motor function declines and incident frailty: A population-based cohort study

**DOI:** 10.3389/fpsyt.2022.939310

**Published:** 2022-10-26

**Authors:** Yang Wang, Chenglong Li, Yanjun Ma, Fanfan Zheng, Wuxiang Xie

**Affiliations:** ^1^Department of Prevention and Health Care, Hospital of Health Science Center, Peking University, Beijing, China; ^2^Peking University Clinical Research Institute, Peking University First Hospital, Beijing, China; ^3^PUCRI Heart and Vascular Health Research Center at Peking University Shougang Hospital, Beijing, China; ^4^Key Laboratory of Molecular Cardiovascular Sciences, Peking University, Ministry of Education, Beijing, China; ^5^School of Nursing, Peking Union Medical College, Chinese Academy of Medical Sciences, Beijing, China

**Keywords:** physical activity, trajectory, incident frailty, group-based trajectory modeling, grip strength, gait speed, chair rise

## Abstract

**Background:**

Maintaining physical function and delaying frailty are of significant importance in both quality of life and health longevity for successful aging. The objective of this study is to investigate whether different trajectories of long-term physical activity (PA) participation are associated with subsequent motor function declines and incident frailty in middle-aged and elderly adults.

**Materials and methods:**

Data from 8,227 aged ≥ 50 years adults enrolled in the English Longitudinal Study of Aging were analyzed. Long-term PA participation trajectories were assessed using group-based trajectory modeling over the first 6-year period from wave 1 (2002–2003) to wave 4 (2008–2009). The longitudinal associations of PA trajectories with motor function declines and incident frailty were evaluated by a linear mixed model and Cox regression model, respectively, with follow-up of 10 years from wave 4 to wave 9 (2018–2019).

**Results:**

Five distinct trajectories of long-term PA participation were identified in the aging cohort, including persistently low-active trajectory (*N* = 2,039), increasing active trajectory (*N* = 1,711), declining active trajectory (*N* = 216), persistently moderate-active trajectory (*N* = 2,254), and persistently high-active trajectory (*N* = 2,007). Compared with the persistently low-active group, the participants in persistently moderate- and high-active groups experienced significantly decelerated grip strength decline, decreased gait speed decline, and faster chair rises after multiple-adjustment. Similarly, participants maintaining moderate- and high-active PA were also associated with a lower risk of incident frailty (multiple-adjusted hazard ratio: 0.70, 95% confidence interval: 0.62–0.80, and 0.42, 95% CI: 0.36–0.49, respectively), compared with those with persistently low PA. Notably, the participants with the increasing active trajectory got similar health benefits as those with persistently moderate and high levels of PA.

**Conclusion:**

In addition to persistent PA, increasing PA was linked to a slower decline in motor function and lower risk of incident frailty in the cohort. Our findings suggest that regular PA is never too late.

## HIGHLIGHTS

- Trajectories of long-term participation in physical activity can be varied among middle and older adults.

- Persistently moderate-, high-active, and increasing participation in physical activity were associated with decelerated motor function decline and reduced risk of incident frailty.

- Strategies focusing on maintaining or increasing active physical activity might promote healthy aging.

- It’s never too late to start or enhance regular physical activity for middle-aged and elderly adults.

## Introduction

The world’s population aged 60 years or older will increase from 841 million in 2013 to more than two billion by 2050 (1). The aging process is characterized by a decline in functional performance and an increase in morbidity, which, although they are two separate conditions, are both related to disability. The total sum of global years lived with disability increased from 562 million to 853 million from 1990 to 2017 (2). Impaired physical functions, such as muscle strength, balance, and gait performance deficits, are common underlying traits among disability. The transition from robustness to disability is a long-lasting, continuous and insidious process that may take years (3). A stage in the transition is referred to as frailty characterized by a decline in functioning across multiple physiological systems and accompanied by an increased vulnerability to stressors (4, 5). Frailty is multidimensional, with physical and psychosocial factors playing a part in its development, an extreme consequence of the normal aging process and dynamic, which means that an individual can fluctuate between states of severity of frailty (6). It is potentially preventable, up to a probable point of no return when it becomes a pre-death phase with profound implications for clinical practice and public health (6). Growing evidence shows that frailty increases the risks of falls, hospitalization, admission to long-term care and mortality (4, 5). In clinical practice, frailty can help clinicians identify patients who may benefit from aggressive interventions and those who might suffer harm from them (7). Together, maintaining function and delaying frailty are of significant importance in both quality of life and longevity.

Increasing evidence reports the benefits yielded by regular physical activity (PA) on the motor function in older people by preserving mobility, muscle strength, and balance (8–10). However, there is a methodological limitation that PA are evaluated at single time-point (primarily the baseline level) or short time-scales without considering the long-term dynamic nature of PA behavior. Group-based trajectory modeling (GBTM) allows grouping of subjects presenting with similar baseline values and longitudinal patterns of change according to their direction and magnitude (11). Using this method, some studies have detected different PA trajectories among older adult cohorts (12–14). Three studies examined the association of PA trajectories with mortality in older adults (15–17). One study investigated the relationship between PA trajectories with contemporaneous physical performance changes in older men (18). But, there isn’t an investigation of the temporal association of long-term PA participation trajectories with subsequent motor function changes and incident frailty.

Therefore, the main objectives of this study were to investigate different trajectories of long-term PA participation over a 6-year span by the GBTM and evaluate their associations with subsequent motor function decline and incident frailty in middle-aged and elderly adults. Our hypotheses are that older adults maintaining PA over time will have a slower motor function decline and a lower risk of incident frailty compared with persistently inactive subjects or those reducing PA levels, and that increasing PA even at older ages promotes healthy aging characterized by reduced motor function decline and incident frailty.

## Materials and methods

### Study population

The English Longitudinal Study of Ageing (ELSA) is a biennial, ongoing prospective, nationally representative cohort study of community-based English adults aged 50 years and older, which collected data in 2002–2003 (wave 1) and follow-up assessments were conducted until 2018–2019 (wave 9). Briefly, this is a population-based prospective cohort study examining the determinants and consequences of frailty. Detailed design and methods of the study have been published previously (19).

**FIGURE 1:**
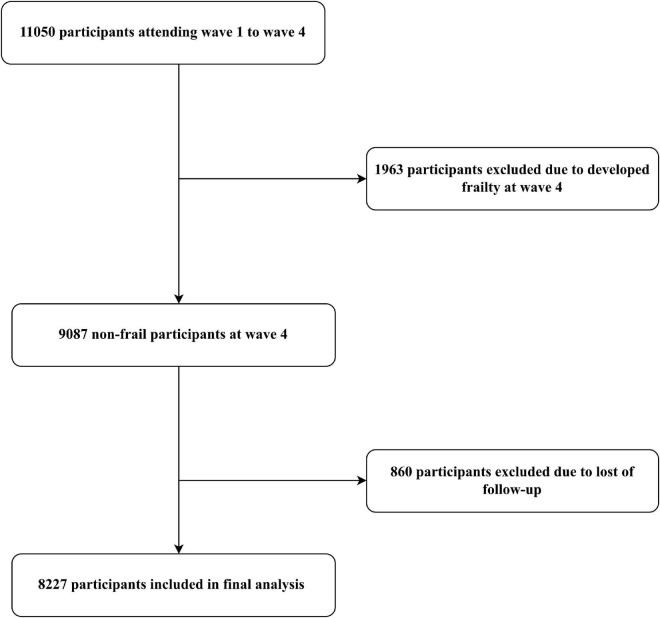
Flow chart of participants selection.

Data from wave 1 to wave 4 (2008–2009) were analyzed to evaluate PA participation trajectories, and wave 4 to wave 9 to assess incident frailty and changes of motor function. Wave 4 was the baseline assessment of frailty and motor function. Excluded participants were those who: (1) developed frailty at wave 4; (2) failed to follow-up from wave 4 to wave 9. Participant selection process of the present study was described in Figure 1.

The ELSA was approved by the London Multicenter Research Ethics Committee (MREC/01/2/91). All participants provided written informed consent.

### Physical activity assessments

The frequency of mild, moderate and vigorous PA was measured by self-reported questionnaire in the ELSA, with a card describing different types of activities presented for participants when being asked about these questions. Further details are provided in the Supplementary methods.

The scoring system for assessing PA consisted of the three-stage approach in the present study. Firstly, the frequency of participating in mild, moderate and vigorous activities was assigned a score of 1 (hardly ever or never), 2 (one to three times per month), 3 (at least once per week), respectively (20). Secondly, the standardized Z scores of assigned scores were generated by subtracting the corresponding mean and dividing by standard deviation (SD), separately. Lastly, a weighted global activity Z scores were calculated to evaluate different intensities of PA. The weights were chosen based on metabolic equivalent of tasks (MET) (21). After calculation, mild, moderate, and vigorous intensity of PA were assigned MET weights of 2.3, 4.4, and 7.2, respectively. Supplementary Table 1 provides the detailed information for MET weights calculation that agreed with previous studies (22).

### Assessment of incident frailty

The frailty index based on an accumulation of age-related deficits and was generated according to a standard procedure (23). Deficits in health were included in the frailty index in the present study if they matched the following criteria: the deficit represented multiple physiological systems; the prevalence of deficits increased with age; the deficit was not too universal in middle-age and the prevalence of the deficit should not be less than 1%.

After screening baseline data of the ELSA, 32 variables were selected to calculate the frailty index, including diseases (based on self-reports or physical measurements), disability in activities of daily living (ADL) and signs of psychological unhealthy (Supplementary Table 2). All variables were recoded, with “0” and “1” indicating the absence and the presence of a deficit, respectively. An additional value of “0.5” for variables with an intermediate response (e.g., “sometimes” or “suspect”) was added. The frailty score was generated for each participant according to the number of deficits present in a person divided by the 32 deficits considered. The frailty score was a continuous variable ranging from 0 to 1, with greater scores indicating higher degree of frailty. The frailty score ≥ 0.25 had been proposed to indicate frailty, with reference to previous studies (6).

### Assessment of motor function

Grip strength was measured three times for each hand using a Smedley dynamometer in the ELSA, and the maximum measurements were used in our analyses. The measurement method of grip strength is consistent with the recent similar study (24). In addition, some studies have found similar test-retest reliability with the mean of two or three trials and the maximum of three trials (25).

Gait speed was measured by asking the participants to walk at their usual pace and use any assistive devices at an 8 ft (2.44 m) marked course. The mean of two trails (m/s) was calculated. The method of measuring gait speed is consistent with previous research (26).

The time (in seconds) it takes to get up and sit down is known as the timed chair rise. Participants sit in a chair and cross their arms over the chest with feet resting on the floor. They were asked to stand up and sit down five times as quickly as possible without using their arms. The complete time was recorded in the five-repetition chair stand test (CS-5). According to previous research, chair rises measurement method is valid (26).

The changes in grip strength, gait speed and timed five chair rises from wave 4 to wave 9 (2002–2003 to 2018–2019) were taken as dependent variables.

### Covariates

Covariates included demographic and health factors were collected at wave 4. The covariates were selected on basis of potential cofounders known to be related with frailty and motor function, including demographic factors (age, sex, ethnicity, educational background, cohabitation status), health behaviors (current smoking, alcohol consumption, and vigorous exercise), and health condition (hypertension, diabetes, stroke, cardiovascular diseases, cancer, chronic lung diseases, depressive symptoms, functional limitations, body mass index [BMI], and physical function measurements). The detailed information of covariates was described in the Supplementary methods.

### Statistical analysis

The GBTM was performed by the SAS Proc Traj procedure to distinguish potential trajectories of long-term PA participation based on weighted Z scores of PA across a 6-year period from waves 1 to 4 in the ELSA. The GBTM used maximum likelihood estimation to identify participants sharing similar trajectory of weighted Z scores, which can process data distributions including censored normal, Poisson and Bernoulli, and was an appropriate option when handling non-monotonic trajectories (27). Then, the most appropriate trajectory group was determined for each participant, which was included in further multivariate analysis as the independent variable. In the Supplementary methods, a detailed description of trajectory modeling was presented.

For the primary analysis, linear mixed models were used to assess the longitudinal associations between PA trajectories and subsequent motor function changes, with the intercept and slope of time fitted as random effects to account for inter-individual differences. Considering capability of linear mixed models in handling response data missing at random, we didn’t implement imputation methods. The longitudinal association between PA trajectories and incident frailty was evaluated using proportional hazard regression (Cox regression) model. Detailed descriptions of the statistical models were provided in the Supplementary methods.

In addition, an exploratory analysis was performed separately in the mild, moderate and vigorous intensity PA trajectories to find out the influence of different intensity PA on the primary results. Several sensitivity analyses were carried out to examine the robustness of the conclusions of the primary analysis. To address reverse causation, the participants who reported difficulties with daily life activities such as bathing, dressing, eating, getting in/out of bed and walking across a room were excluded, during waves 1 to 4.

All statistical analyses were conducted using SAS 9.4 (SAS Institute, Cary, NC, United States). A two-tailed alpha of 0.05 was referred to as statistically significant level.

## Results

### Baseline characteristics

A total of 8,227 frailty-free participants from the ELSA were included in the analysis. Detailed description of participant selection was shown in Figure 1. Of the included participants, the mean age was 57.8 ± 9.2 years and 45.9% were male.

### Physical activity participation trajectories from wave 1 to 4

**FIGURE 2:**
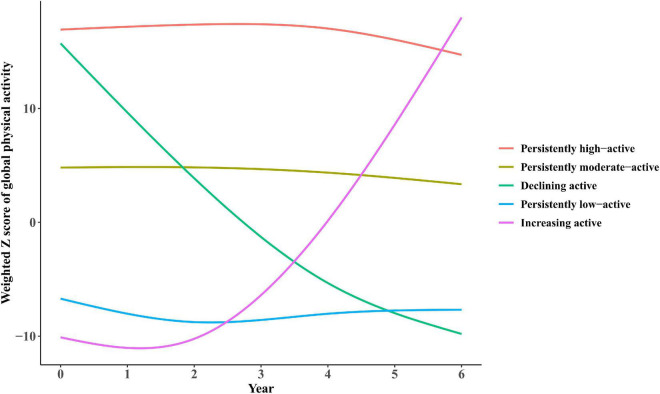
Trajectories of participation in global physical activities by participants from the ELSA over a 6-year span. (1) Persistently low-active trajectory (*N* = 2,039), representing low participation in physical activity (PA) during waves 1–4; (2) increasing active trajectory (*N* = 1,711), representing initially low participation in PA but turned to elevating afterward; (3) declining active trajectory (*N* = 216), representing high participation at early stages but turned to decreasing afterward; (4) persistently moderate-active trajectory (*N* = 2,254), representing constantly moderate participation; (5) persistently high-active trajectory (*N* = 2,007), representing highly participation.

Five trajectories of long-term PA participation based on weighted activity Z scores were identified from waves 1 to 4 of the ELSA. The five trajectories were shown in Figure 2, including: (1) persistently low-active trajectory (*N* = 2,039), representing low participation in PA during waves 1 to 4; (2) increasing active trajectory (*N* = 1,711), representing initially low participation in PA but turned to elevating afterward; (3) declining active trajectory (*N* = 216), representing high participation at early stages but turned to decreasing afterward; (4) persistently moderate-active trajectory (*N* = 2,254), representing constantly moderate participation; (5) persistently high-active trajectory (*N* = 2,007), representing highly participation.

Table 1 displays the baseline characteristics of the study participants by five PA trajectories. Compared with the other PA trajectory groups, participants in the persistently high-active and increasing active trajectory groups were more likely to be men, had lower BMI, frailty index and better motor function performance.

**TABLE 1:** Baseline characteristics of study population according to physical activity trajectories.

Characteristics^a^	All *N* = 8,227	Increasing active *N* = 1,711	Persistently low *N* = 2,039	Declining active *N* = 216	Persistently moderate *N* = 2,254	Persistently high *N* = 2,007	*P-value* ^b^
Male (%)	3,780 (45.9%)	872 (51.0%)	872 (42.8%)	90 (41.7%)	949 (42.1%)	997 (49.7%)	<0.001
Age (years)	57.8 ± 9.2	52.1 ± 7.5	56.2 ± 9.8	64.0 ± 9.6	61.6 ± 8.8	59.4 ± 7.5	<0.001
White (%)	7,945 (96.6%)	1,635 (95.6%)	1,919 (94.1%)	207 (95.8%)	2,205 (97.8%)	1,979 (98.6%)	<0.001
High level education (%)	2,810 (34.2%)	585 (34.2%)	389 (19.1%)	60 (27.8%)	779 (34.6%)	997 (49.7%)	<0.001
Living alone (%)	1,400 (17.0%)	39 (2.3%)	197 (9.7%)	64 (29.6%)	637 (28.3%)	463 (23.1%)	<0.001
Current smoking (%)	757 (9.2%)	26 (1.5%)	116 (5.7%)	36 (16.7%)	362 (16.1%)	217 (10.8%)	<0.001
Drinking ≥ once per week (%)	3,353 (40.8%)	84 (4.9%)	274 (13.4%)	125 (57.9%)	1,407 (62.4%)	1,463 (72.9%)	<0.001
Depressive symptoms (%)	496 (6.0%)	15 (0.9%)	86 (4.2%)	30 (13.9%)	245 (10.9%)	120 (6.0%)	<0.001
Hypertension (%)	2,545 (30.9%)	62 (3.6%)	329 (16.1%)	117 (54.2%)	1,156 (51.3%)	881 (43.9%)	<0.001
Diabetes (%)	209 (2.5%)	2 (0.1%)	35 (1.7%)	10 (4.6%)	101 (4.5%)	61 (3.0%)	<0.001
Stroke	91 (1.1%)	2 (0.1%)	25 (1.2%)	5 (2.3%)	39 (1.7%)	20 (1.0%)	<0.001
Cardiovascular disease (%)	353 (4.3%)	16 (0.9%)	62 (3.0%)	22 (10.2%)	153 (6.8%)	100 (5.0%)	<0.001
Chronic lung disease (%)	168 (2.0%)	6 (0.4%)	28 (1.4%)	12 (5.6%)	80 (3.5%)	42 (2.1%)	<0.001
Cancer (%)	239 (2.9%)	5 (0.3%)	27 (1.3%)	13 (6.0%)	96 (4.3%)	98 (4.9%)	<0.001
BMI (kg/m^2^)	27.7 ± 4.8	27.4 ± 4.6	28.6 ± 5.3	28.2 ± 4.9	27.9 ± 4.7	27.0 ± 4.3	<0.001
Frailty index	0.07 (0.04–0.13)	0.05 (0.03–0.10)	0.10 (0.05–0.16)	0.12 (0.06–0.18)	0.08 (0.04–0.14)	0.05 (0.02–0.09)	<0.001
Grip strength (kg)	33.0 ± 11.2	36.5 ± 11.3	31.7 ± 11.3	27.9 ± 10.5	30.7 ± 10.5	34.5 ± 10.8	<0.001
Gait speed (cm/s)	98.4 ± 28.0	103.8 ± 28.7	90.3 ± 27.5	82.0 ± 23.4	95.5 ± 26.6	106.8 ± 27.4	<0.001
Timed 5 chair rises (s)	10.9 ± 3.6	9.8 ± 2.9	11.4 ± 4.0	12.4 ± 3.9	11.6 ± 3.7	10.4 ± 3.2	<0.001

^a^Data are presented as mean ± SD, *n* (%), or median (quartile 1–quartile 3). ^b^*P***-**value reported for differences between trajectory groups using analysis of variance, chi-square test, or Kruskal-Wallis test.

### Physical activity participation trajectories and subsequent motor function changes

The longitudinal associations of the PA trajectories with subsequent motor function changes are shown in Table 2. After multiple-adjustment, the participants with persistently moderate and high PA trajectories experienced decelerated grip strength decline (β = 0.999 kg, 95% confidence interval [CI]: 0.314–1.684, *P* = 0.004; β = 1.334 kg, 95% CI: 0.641–2.026, *P* < 0.001), decreased gait speed decline (β = 4.655 cm/s, 95% CI: 1.582–7.727, *P* = 0.003; β = 7.949 cm/s, 95% CI: 4.783–11.116, *P* < 0.001), and faster chair rises (β = –0.519 s, 95% CI: –0.812 to –0.226, *P* = 0.001; β = –0.904 s, 95% CI: –1.199 to –0.608, *P* < 0.001) compared with participants with the persistently low-active trajectory. Notably, the increasing active trajectory was associated with subsequent motor function comprehensive improvement (grip strength: β = 1.799 kg, 95% CI: 1.009–2.589, *P* < 0.001; grip speed: β = 5.012 cm/s, 95% CI: 1.475–8.548, *P* = 0.005; timed five chair rises: β = –0.904 s, 95% CI: –1.199 to –0.608, *P* < 0.001), while the longitudinal associations of the declining active trajectory with motor function changes were not found.

**TABLE 2:** Mean differences of changes in physical function between global physical activity trajectories.

Global physical activity trajectories	Grip strength (kg)		Gait speed (cm/s)		Timed 5 chair rises (s)	
	β (95% Cl)^a^	*P-value*	β (95% Cl)^a^	*P-value*	β (95% Cl)^a^	*P-value*
Persistently low-active	Reference		Reference		Reference	
Increasing active	1.799 (1.009, 2.589)	<0.001	5.012 (1.475, 8.548)	0.005	–0.513 (–0.789, –0.236)	<0.001
Declining active	0.785 (–0.683, 2.253)	0.295	2.789 (–4.083, 9.662)	0.426	–0.072 (–0.727, 0.582)	0.829
Persistently moderate-active	0.999 (0.314, 1.684)	0.004	4.655 (1.582, 7.727)	0.003	–0.519 (–0.812, –0.226)	0.001
Persistently high-active	1.334 (0.641, 2.026)	<0.001	7.949 (4.783, 11.116)	<0.001	–0.904 (–1.199, –0.608)	<0.001

^a^Adjusted for age, sex, ethnicity, education, cohabitation status, current smoking, alcohol consumption, depressive symptoms, hypertension, diabetes, stroke, cardiovascular diseases, chronic lung diseases, cancer, functional limitations, BMI, and physical function measurements at wave 4.

Further analyses were conducted in the mild, moderate and vigorous intensity PA (Supplementary Table 3). Similar to global PA trajectories, five trajectories were identified for mild and moderate intensity PA, while four trajectories were identified for vigorous intensity PA, as shown in Supplementary Figures 1–3. The subgroup analyses found similar results to the global PA results, although the significant association was mainly observed in the vigorous intensity PA.

### Physical activity participation trajectories and subsequent incident frailty

There were 1,866 incidents of frailty during a 10-year follow-up period, data summarized in Table 3. In contrast to subjects with the persistently low-active trajectory, participants with persistently moderate and high trajectories had a significant lower risk of incident frailty, with a multivariate-adjusted HR of 0.70 (95% CI: 0.62–0.80, *P* < 0.001) and 0.42 (95% CI: 0.36–0.49, *P* < 0.001), respectively (Table 3). Importantly, similar to the longitudinal associations of PA trajectories with motor function changes, participants with increasing active trajectory also had a significantly lower risk of incident frailty (HR = 0.60, 95% CI: 0.50–0.72, *P* < 0.001), while the association of the declining active trajectory with the risk of incident frailty was not identified (Table 3). The subgroup analyses with different intensities of PA remain generally consistent with our primary findings (Supplementary Table 4).

**TABLE 3:** Hazard ratios and 95% confidence intervals of incident frailty by global physical activity trajectories.

Global physical activity trajectories	Events/Total	Risk for incident frailty^a^	
		HR (95% CI)	*P-value*
Persistently low-active	596/2,039	Reference	
Increasing active	202/1,711	0.60 (0.50, 0.72)	<0.001
Declining active	89/216	0.93 (0.73, 1.17)	0.527
Persistently moderate-active	668/2,254	0.70 (0.62, 0.80)	<0.001
Persistently high-active	311/2,007	0.42 (0.36, 0.49)	<0.001

^a^Adjusted for age, sex, ethnicity, education, cohabitation status, current smoking, alcohol consumption, depressive symptoms, hypertension, diabetes, stroke, cardiovascular diseases, chronic lung diseases, cancer, functional limitations, and BMI.

### Sensitivity analyses

According to the sensitivity analyses that excluded participants who reported daily activities difficulties during waves 1–4, the associations of PA trajectories with motor function declines and incident frailty remained the same as those from the main analyses (Supplementary Tables 5, 6). Besides, “The subgroup analyses based on gender and age range were performed. Findings in subgroup analyses were consistent with the results of previous analyses (Supplementary Tables 8–15).”

## Discussion

By following a longitudinal cohort of 8,227 middle-aged and older adults from the ELSA, we identified five distinct trajectories of long-term PA participation over a 6-year span and observed prospective associations of PA trajectories with subsequent 10-year incident frailty and motor function performance declines, including grip strength, gait speed and timed five chair rises. Compared with those in the persistently low-active group, individuals in all other trajectories, except for those with the declining active trajectory, had slower motor function declines and a lower risk of incident frailty. Importantly, individuals with an increasing active trajectory had similar or even lower physical performance declines and risk of incident frailty than those with a persistently moderate or high active trajectory. Our findings are encouraging, not confined to participants with persistently active participation in PA, who can still gain substantial benefits in the quality of life by becoming more physically active irrespective of past physical activity levels, providing further evidence to the broad public health benefits of PA. In clinical practice, the elderly is encouraged to exercise regularly to reduce the incidence of frailty that help clinicians identify patients who may benefit from aggressive interventions and increases the risks of falls, hospitalization, admission to long-term care and mortality (4, 5). To our knowledge, this is the first study to evaluate temporal associations of long-term PA participation trajectories with subsequent motor function declines and incident frailty in middle-aged and elderly adults.

Previous studies on the relationship between long-term PA participation trajectories and motor function changes are lack of prospectiveness and applied to some certain populations (18, 28). Laddu et al. identified three PA groups using the GBTM approach only in older men and demonstrated that men in moderate and high activity trajectory groups had contemporaneous higher performance outcomes and experienced smaller declines in nearly each performance outcome than men in the low activity trajectory group (18). Pettee Gabriel et al. used latent class growth analysis, identified five PA trajectory groups only in middle-aged women and found that women included in the middle and highest physical activity groups demonstrated ≥ 5% better physical functioning performance in late midlife than those who maintained low physical activity levels (28). Despite different approaches, we both demonstrated five similar trends in PA trajectory and found that increasing active participation in PA could reduce the rate of physical performance declines, even for those who were physically inactive at an early age. But neither of the previous studies could verify the temporal correlation between PA trajectories and the physical performance declines. With the prospective design, we confirm and expand upon observations by Pettee Gabriel et al. (28) and other investigators (18, 29–31) by prospectively assessing associations of PA trajectories with subsequent motor changes in men and women aged 50 years and older. And our findings also provide encouraging information that individuals with increasing active participation in PA had minimal and comparable rates of motor function declines to those with persistently active participation in PA. Our findings suggest that regular physical activity is never be too late.

In the current study, gait speed, grip strength, and timed five chair rises were used as indicators of motor function. Gait speed has been considered to be an invaluable health functional vital sign and a core indicator of many health outcomes especially mortality and disability (32, 33). Although the decline of gait speed in this study didn’t reach the risk-effect reduction of 10 cm/s confirmed by the systematic review of association between gait speed with mortality and cardiovascular disease (33), the differences observed among the PA trajectories in this study met a threshold of meaningful change in gait speed (4–6 cm/s) (34, 35). Slower gait speed declines may reflect the enhancement of multi-system organ function by being physically active. The grip strength can represent global muscle strength in older people in the community (36). Of note, faster grip strength decline may reveal immediacy of death rather than age-related decline, and be more accurate at detecting the risk of mortality in very old adults (37, 38). Peterson et al. found that each 0.1 decrease in normalized grip strength was associated with a 15 and 12% increased risk of mortality in older Mexican American men and women, respectively (39). The chair rise test can be commonly used as a proxy for the power and strength of the lower limbs. The results of a previous study indicated that participants who performed poorly on the timed five chair rise test at baseline were at a significantly higher risk of developing disability (40). Additionally, it is one of the preferred ways to assess physical function in sarcopenia (41). The effect of regular physical activity on these meaningful indicators of motor function suggests the health benefits of regular physical activity.

Physical activity is known to preserve or improve physical function and delay or reverse frailty (42). Data from UK cohort study showed that active participants had a lower risk of frailty compared with inactive participants (43). Borda et al. also reported that PA was significantly associated with a lower risk of developing frailty in the Mexican Health and Aging Study cohort (44). Based on mobile healthcare and wearable technologies, Li et al. found that for 1 SD decrease in the temporal activity (more random activity fluctuations) correlations the risk of frailty increased by 31%; the risk of disability increased by 15–25%; and the risk of death increased by 26% (45). More recently, Yamada et al. found that older adults are more likely to experience incident frailty/disability due to decreased PA during the COVID-19 pandemic (46). Although previous studies have advanced our knowledge regarding PA and incident frailty, there isn’t an investigation of the temporal association between long-term PA trajectories and incident frailty, thus providing an incomplete picture of the total evidence base on this subject. The current study confirmed previous findings and provided new evidence for a temporal association between long-term PA participation trajectories and subsequent incident frailty. This study showed that lower risks of incident frailty were obtained not only by persistently moderate or high active older adults but also by those who become more physically active regardless of their baseline level.

Our study has several strengths that make our findings more valid and robust. First, we prospectively assessed a temporal association of PA trajectories with subsequent motor function declines and incident frailty in a relatively large and nationally-representative sample with a long follow-up of 10 years. Such a prospective cohort design improves the ability to draw casual conclusions. Second, we explored all possible patterns of PA over the span of 6 years using the novel GBTM method that could address limitations of only considering activity participation at a single time point by efficiently incorporating measurements of activity participation at multiple time points. Finally, we used validated measures for assessing motor function and replicated results with relatively small heterogeneity by controlling confounding factors and sensitivity analysis.

Notwithstanding these strengths, our study has several limitations. Firstly, the ELSA’s regional basis may limit our ability to generalize our results across ethnically diverse populations. Secondly, we used self-reported PA frequency when evaluating PA trajectories, thus recall errors are inevitable especially in older adults. Over-reporting of PA levels, if present, would lead to an underestimation of the actual effect of PA (47). In addition, we did not consider duration of PA when assessing PA trajectories. Thirdly, a total of 2,823 (25.55%) individuals were excluded from the analysis due to loss during follow up or develop frailty at wave 4. Individuals who were excluded were older and had higher percentages of living alone and higher prevalence of chronic diseases at baseline, compared with included older adults (Supplementary Table 7). Hence, selection bias could restrict the generalizability of our results. Fourthly, GBTM method inherently simplifies the variability of individual trajectories within classes. Individuals may show more change than others within the same group, and conclusions about individual outcomes should be approached with caution (11). Fifthly, our study is based on observation, and reverse causality cannot be excluded. PA trajectories may be influenced by health status. To address the issue, we performed sensitivity analyses in that participants reported daily activities difficulties during waves 1–4 were further excluded and observed consistent results. Finally, there may still be confounding bias due to unmeasured confounding factors such as pro-inflammatory cytokine interleukin-6 (48).

## Conclusion

In summary, this study demonstrated that not only persistently active participation in PA, but also gradually improved PA were associated with subsequent a slower decline in motor function and a lower risk of incident frailty in middle-aged and older adults. Strategies focusing on maintaining or increasing PA may promote healthy aging and longevity.

## Data availability statement

Access to original ELSA datasets can be obtained by visiting the website (https://www.elsa-project.ac.uk/).

## Ethics statement

The ELSA was approved by the London Multicenter Research Ethics Committee (MREC/01/2/91). The patients/participants provided their written informed consent to participate in this study.

## Author contributions

FZ and WX: had full access to all the data in the study and took responsibility for the integrity of the data and the accuracy of the data analysis. YW, FZ, and WX: study concept, design, and drafting of the manuscript. YW, CL, FZ, and WX: acquisition, analysis, or interpretation of data. YW, CL, and WX: statistical analysis. WX: obtained funding, administrative, technical, or material support, and supervision. All authors critical revision of the manuscript for important intellectual content and approved the submitted version.
