# Supplementary material for: Associations of physical activity participation trajectories with subsequent motor function declines and incident frailty: A population-based cohort study
Source: Front Psychiatry. 2022 Oct 26;13:939310. doi: 10.3389/fpsyt.2022.939310 (PMC9644212; doi:10.3389/fpsyt.2022.939310)
Supplement: Supplementary file 1 [file Data_Sheet_1.docx]

**Supplementary material online**

Yang Wang, Chenglong Li, Yanjun Ma, Fanfan Zheng, Wuxiang Xie. Associations of physical activity participation trajectories with subsequent motor function declines and incident frailty: a population-based cohort study

**eMethods**

[**eTable 1.** Physical activity intensity categories, activity types, MET values, codes, and MET weights](#_Toc115364046)

[**eTable 2.** The components of constructed 32-item frailty index.](#_Toc115364047)

[**eTable 3.** Mean differences of changes in physical function between sub-domain physical activity trajectories.](#_Toc115364048)

[**eTable 4.** Hazard ratios and 95% confidence intervals of incident frailty by sub-domain physical activity trajectories.](#_Toc115364049)

[**eTable 5.** Mean differences of changes in physical function between global physical activity trajectories, further excluding participants reported daily activities difficulties during waves 1 to 4.](#_Toc115364050)

[**eTable 6.** Hazard ratios and 95% confidence intervals of incident frailty by global physical activity trajectories, further excluding participants reported daily activities difficulties during waves 1 to 4.](#_Toc115364051)

[**eTable 7.** Non-response analysis comparing baseline characteristics between included and excluded participants of the ELSA.](#_Toc115364052)

[**eTable 8.** Mean differences of changes in physical function between global physical activity trajectories among men.](#_Toc115364053)

[**eTable 9**. Mean differences of changes in physical function between global physical activity trajectories among women.](#_Toc115364054)

[**eTable 10.** Hazard ratios and 95% confidence intervals of incident frailty by global physical activity trajectories among men.](#_Toc115364055)

[**eTable 11.** Hazard ratios and 95% confidence intervals of incident frailty by global physical activity trajectories among women.](#_Toc115364056)

[**eTable 12.** Mean differences of changes in physical function between global physical activity trajectories among people aged < 60 years.](#_Toc115364057)

[**eTable 13**. Mean differences of changes in physical function between global physical activity trajectories among people aged ≥ 60 years.](#_Toc115364058)

[**eTable 14.** Hazard ratios and 95% confidence intervals of incident frailty by global physical activity trajectories among people aged < 60 years.](#_Toc115364059)

[**eTable 15.** Hazard ratios and 95% confidence intervals of incident frailty by global physical activity trajectories among people aged ≥ 60 years.](#_Toc115364060)

[**eFigure 1.** Trajectories of participation in mild intensity physical activities by participants from the ELSA over a 6-year span.](#figure2)

[**eFigure 2.** Trajectories of participation in moderate intensity physical activities by participants from the ELSA over a 6-year span.](#figure3)

[**eFigure 3.** Trajectories of participation in vigorous intensity physical activities by participants from the ELSA over a 6-year span**.**](#figure4)

**eMethods**

**Physical activity assessment**

The ELSA used three questions to measure participation in physical activities of mild, moderate and vigorous intensity. Unified expression of these questions was “We would like to know the type and amount of physical activity involved in your daily life. Do you take part in sports or activities that are vigorous/moderately energetic/mildly energetic more than once a week, or once a week, or one to three times a month, or hardly ever or never?”. A card was presented for participants when being asked about these questions, with examples of different types of activities provided. Detailed examples included: 1) laundry, home repairs for mild intensity; 2) gardening, cleaning the car, walking at a moderate pace, dancing, floor or stretching exercises for moderate intensity; 3) running or jogging, swimming, cycling, aerobics or gym workout, tennis, digging with a spade or shovel for vigorous intensity. Participants were also allowed to list additional examples and required to decide which of the three categories (vigorous, moderate and mild) could best match the activity.

**Statistical analysis**

The GBTM can fit non-monotonic trajectories and support multiple trajectory shapes including linear, quadratic and cubic. It also allows specification of number of trajectory groups before fitting the model. We selected number of groups from 3 to 7 and compared model fit statistics of the Bayesian information criterion (BIC) of different trajectory models to determine the most optimal number of trajectory groups. Then, we determined that modeling 5 trajectory groups was appropriate for global, mild and moderate physical activity trajectories modeling, while 4 trajectory groups for vigorous physical activity trajectories modeling.

We further evaluated different trajectory shapes for each trajectory group by testing the null hypothesis that the shape parameter for the group equals zero. We also used graphics of trajectory group means to help determine which shape best fit each trajectory group. After the procedure, we determined that the best 5-group trajectory model for global, mild and moderate physical activity consisted of 2 cubic and 3 linear trajectories, while the best 4-group model for vigorous physical activity consisted of 3 cubic and 1 linear trajectories. Then the estimated trajectory groups membership was included as the independent variable for further multivariate analysis.

Association between physical activity trajectories and incident frailty was evaluated using proportional hazard regression (Cox regression) model. We evaluated proportional hazard assumption for all included covariates using weighted Schoenfeld residuals, and addressed violation of assumption by including covariates as interaction term with time scale variable (years from wave 4 to first occurrence of event in interest or censoring). After assessment, no significant violation was identified.

**eTable 1.** Physical activity intensity categories, activity types, MET values, codes, and MET weights

| **Intensity** | **Activity type ^a^** | **MET ^b^** | **Code ^c^** | **MET weight ^d^** |
| --- | --- | --- | --- | --- |
| Low | Laundry | 2.15 | Mean of 05090, and 05095 | 2.3 |
| Low | Home repairs | 2.50 | 06126 |  |
| Moderate | Gardening | 3.80 | 08245 | 4.4 |
| Moderate | Cleaning the car | 3.50 | 05020 |  |
| Moderate | Walking at a moderate pace | 5.30 | 17082 |  |
| Moderate | Dancing | 5.85 | Mean of 03010, 03014, 03019, 03020, 03025, 03030, 03031, 03038, 03040,03050, 03060 |  |
| Moderate | Floor or stretching exercises | 3.50 | 05130 |  |
| Vigorous | Running or jogging | 6.70 | Mean of 12010, 12020, 12025, 12027, 12150 | 7.2 |
| Vigorous | Swimming | 7.20 | Mean of 18230, 18240, 18310 |  |
| Vigorous | Cycling | 6.80 | 01011 |  |
| Vigorous | Aerobics or gym workout | 7.30 | 03015 |  |
| Vigorous | Tennis | 7.10 | Mean of 15675, 15680, 15690 |  |
| Vigorous | Digging with a spade or shovel | 7.80 | 08052 |  |

^a^ Activity type was selected based on examples showed to participants when investigating frequency of physical activity.

^b^ MET: metabolic equivalent of tasks. MET estimates were derived according to 2011 Compendium of Physical Activities.

^c^ Code represented exact type of activities, for activity corresponding to multiple potential types, we used mean of MET values from these activities. ^d^ Mean of all activity types’ MET was used to calculate MET weights for low, moderate, and vigorous intensity activities, respectively.

**eTable 2.** The components of constructed 32-item frailty index.

| **Item number ^a^** | **Item definition ^b^** | **Scoring ^c^** |
| --- | --- | --- |
| 1 | Self-reported difficulties in bathing because of a physical, mental, emotional or memory problem. | Yes=1.00; No=0.00 |
| 2 | Self-reported difficulties in dressing because of a physical, mental, emotional or memory problem. | Yes=1.00; No=0.00 |
| 3 | Self-reported difficulties in getting in/out of bed because of a physical, mental, emotional or memory problem. | Yes=1.00; No=0.00 |
| 4 | Self-reported difficulties in walking around the house because of a physical, mental, emotional or memory problem. | Yes=1.00; No=0.00 |
| 5 | Self-reported difficulties in eating because of a physical, mental, emotional or memory problem. | Yes=1.00; No=0.00 |
| 6 | Self-reported difficulties in finishing daily activities of using the toilet because of a physical, mental, emotional or memory problem. | Yes=1.00; No=0.00 |
| 7 | Total number (0-5) of self-reported difficulties in finishing daily activities including bathing, dressing, eating, getting in/out of bed, walking around the house. | Any difficulties=1.00; No difficulties=0.00 |
| 8 | Self-reported difficulties in housework because of a physical, mental, emotional or memory problem. | Yes=1.00; No=0.00 |
| 9 | Self-reported difficulties in shopping because of a physical, mental, emotional or memory problem. | Yes=1.00; No=0.00 |
| 10 | Self-reported difficulties in preparing hot meal because of a physical, mental, emotional or memory problem. | Yes=1.00; No=0.00 |
| 11 | Self-reported difficulties in taking prescribed medications because of a physical, mental, emotional or memory problem. | Yes=1.00; No=0.00 |
| 12 | Self-reported difficulties in managing money because of a physical, mental, emotional or memory problem. | Yes=1.00; No=0.00 |
| 13 | Self-reported difficulties in getting up from a chair because of a physical, mental, emotional or memory problem. | Yes=1.00; No=0.00 |
| 14 | Self-reported difficulties in climbing several flights of stairs because of a physical, mental, emotional or memory problem. | Yes=1.00; No=0.00 |
| 15 | Self-reported difficulties in lifting or carrying weights over 10 pounds because of a physical, mental, emotional or memory problem. | Yes=1.00; No=0.00 |
| 16 | Self-reported difficulties in walking one block because of a physical, mental, emotional or memory problem. | Yes=1.00; No=0.00 |
| 17 | Self-reported rating of health status. | Poor=1.00; Fair=0.75; Good=0.50; Very Good=0.25; Excellent=0.00 |
| 18 | Change in self-reported rating of health status. | Worse=1.00; Better/Same=0.00 |
| 19 | Feeling that everything is an effort much of time. | Yes=1.00; No=0.00 |
| 20 | Feeling depressed much of time. | Yes=1.00; No=0.00 |
| 21 | Feeling happy much of time. | No=1.00; Yes=0.00 |
| 22 | Feeling lonely much of time. | Yes=1.00; No=0.00 |
| 23 | Feeling that could not get going much of time. | Yes=1.00; No=0.00 |
| 24 | Self-reported diagnosis of hypertension by physician. | Yes=1.00; No=0.00 |
| 25 | Self-reported diagnosis of stroke by physician. | Yes=1.00; No=0.00 |
| 26 | Self-reported diagnosis of cancer by physician. | Yes=1.00; No=0.00 |
| 27 | Self-reported diagnosis of diabetes by physician. | Yes=1.00; No=0.00 |
| 28 | Self-reported diagnosis of arthritis by physician. | Yes=1.00; No=0.00 |
| 29 | Self-reported diagnosis of chronic lung disease by physician. | Yes=1.00; No=0.00 |
| 30 | Self-reported diagnosis of heart attack by physician. | Yes=1.00; No=0.00 |
| 31 | Self-reported diagnosis of congestive heart failure by physician. | Yes=1.00; No=0.00 |
| 32 | Cognitive status, based on combination of self-reported diagnosis, and cognition score. | Dementia=1.00; Cognitive impairment but not demented=0.50; Cognitive healthy=0.00 |

^a^ An unified 32-item frailty index was utilized across different waves of the ELSA.

^b^ Item definition was based on self-reported information or objective measurements or both.

^c^ The frailty index was calculated as the sum of scoring divided by the total number of items.

**eTable 3.** Mean differences of changes in physical function between sub-domain physical activity trajectories.

| **Sub-domain physical activity trajectories** | **Hand grip strength (kg)** | |  | **Timed 5 chair rises (s)** | |  | **Gait speed (cm/s)** | |  |
| --- | --- | --- | --- | --- | --- | --- | --- | --- | --- |
|  | **β (95% Cl)^a^** | **P value** |  | **β (95% Cl)** | **P value** |  | **β (95% Cl)** | **P value** |  |
| **Mild intensity physical activity trajectories** | | | | | | | | | |
| Persistently low | Reference | |  | Reference | |  | Reference | |  |
| Initially low then improving | 0.419 (-1.244, 2.082) | 0.621 |  | -0.108 (-0.647, 0.432) | 0.696 |  | -0.153 (-7.031, 6.724) | 0.965 |  |
| Initially high then declining | -0.141 (-2.469, 2.188) | 0.906 |  | -0.627 (-1.718, 0.464) | 0.260 |  | -0.962 (-11.58, 9.662) | 0.859 |  |
| Persistently high | 0.812 (-0.841, 2.465) | 0.336 |  | -0.549 (-1.115, 0.018) | 0.058 |  | 2.371 (-4.606, 9.347) | 0.505 |  |
| Initially improving then declining | 0.068 (-1.966, 2.102) | 0.948 |  | -0.189 (-0.992, 0.613) | 0.644 |  | -0.216 (-9.025, 8.592) | 0.962 |  |
| **Moderate intensity physical activity trajectories** | | | | | | | | | |
| Persistently low | Reference | |  | Reference | |  | Reference | |  |
| Initially low then improving | 1.373 (0.033, 2.713) | 0.045 |  | -0.091 (-0.533, 0.351) | 0.687 |  | -2.004 (-7.589, 3.580) | 0.482 |  |
| Initially high then declining | 1.047 (-0.827, 2.920) | 0.273 |  | 0.027 (-0.794, 0.848) | 0.949 |  | -5.282 (-14.04, 3.474) | 0.237 |  |
| Persistently high | 1.912 (0.603, 3.220) | 0.004 |  | -0.591 (-1.056, -0.126) | 0.013 |  | 2.683 (-2.951, 8.317) | 0.351 |  |
| Initially improving then declining | 0.937 (-0.712, 2.585) | 0.265 |  | -0.476 (-1.155, 0.204) | 0.170 |  | -0.618 (-8.054, 6.817) | 0.871 |  |
| **Vigorous intensity physical activity trajectories** | | | | | | | | | |
| Persistently low | Reference | |  | Reference | |  | Reference | |  |
| Initially low then improving | 1.035 (0.491, 1.579) | <0.001 |  | -0.332 (-0.558, -0.106) | 0.004 |  | 3.586 (1.048, 6.123) | 0.006 |  |
| Initially high then declining | 0.315 (-0.319, 0.949) | 0.330 |  | -0.339 (-0.646, -0.033) | 0.030 |  | 2.169 (-0.901, 5.238) | 0.166 |  |
| Persistently high | 0.689 (0.215, 1.164) | 0.004 |  | -0.669 (-0.903, -0.435) | <0.001 |  | 5.584 (3.292, 7.875) | <0.001 |  |

^a^ Adjusted for age, sex, ethnicity, education, cohabitation status, current smoking, alcohol consumption, depressive symptoms, hypertension, diabetes, stroke, cardiovascular diseases, chronic lung diseases, cancer, functional limitations, BMI, and physical function measurements at wave 4.

**eTable 4.** Hazard ratios and 95% confidence intervals of incident frailty by sub-domain physical activity trajectories.

| **Sub-domain physical activity trajectories** | **Events/Total** | **Risk for incident frailty^a^** | |
| --- | --- | --- | --- |
|  |  | **HR (95% CI)** | **P value** |
| **Mild intensity physical activity trajectories** | | | |
| Persistently low | 73/319 | Reference | |
| Initially low then improving | 520/3027 | 0.84 (0.63, 1.11) | 0.215 |
| Initially high then declining | 39/96 | 0.85 (0.56, 1.30) | 0.453 |
| Persistently high | 1166/4600 | 0.62 (0.46, 0.82) | <0.001 |
| Initially improving then declining | 68/185 | 0.74 (0.51, 1.07) | 0.105 |
| **Moderate intensity physical activity trajectories** | | | |
| Persistently low | 196/545 | Reference | |
| Initially low then improving | 499/2963 | 0.65 (0.54, 0.79) | <0.001 |
| Initially high then declining | 99/195 | 0.78 (0.60, 1.01) | 0.062 |
| Persistently high | 956/4252 | 0.44 (0.37, 0.53) | <0.001 |
| Initially improving then declining | 116/272 | 0.64 (0.50, 0.83) | <0.001 |
| **Vigorous intensity physical activity trajectories** | | | |
| Persistently low | 1172/4049 | Reference | |
| Initially low then improving | 240/1845 | 0.59 (0.51, 0.69) | <0.001 |
| Initially high then declining | 233/791 | 0.86 (0.74, 0.99) | 0.040 |
| Persistently high | 221/1542 | 0.48 (0.41, 0.56) | <0.001 |

^a^ Adjusted for age, sex, ethnicity, education, cohabitation status, current smoking, alcohol consumption, depressive symptoms, hypertension, diabetes, stroke, cardiovascular diseases, chronic lung diseases, cancer, functional limitations, and BMI.

**eTable 5.** Mean differences of changes in physical function between global physical activity trajectories, further excluding participants reported daily activities difficulties during waves 1 to 4.

| **Global physical activity trajectories** | **Hand grip strength (kg)** | |  | **Timed 5 chair rises (s)** | |  | **Gait speed (cm/s)** | |  |
| --- | --- | --- | --- | --- | --- | --- | --- | --- | --- |
|  | **β (95% Cl)** **^†^** | **P value** |  | **β (95% Cl)** | **P value** |  | **β (95% Cl)** | **P value** |  |
| Persistently low | Reference | |  | Reference | |  | Reference | |  |
| Initially low then improving | 1.872 (1.045, 2.699) | <0.001 |  | -0.467 (-0.750, -0.184) | 0.001 |  | 5.026 (1.279, 8.773) | 0.009 |  |
| Initially high then declining | 0.418 (-1.276, 2.113) | 0.628 |  | -0.540 (-1.292, 0.211) | 0.158 |  | 1.752 (-5.973, 9.478) | 0.657 |  |
| Persistently moderate | 0.868 (0.106, 1.630) | 0.025 |  | -0.452 (-0.771, -0.133) | 0.005 |  | 4.069 (0.740, 7.397) | 0.017 |  |
| Persistently high | 1.273 (0.514, 2.032) | 0.001 |  | -0.805 (-1.121, -0.489) | <0.001 |  | 7.228 (3.825, 10.630) | <0.001 |  |

^a^ Adjusted for age, sex, ethnicity, education, cohabitation status, current smoking, alcohol consumption, depressive symptoms, hypertension, diabetes, stroke, cardiovascular diseases, chronic lung diseases, cancer, BMI, and physical function measurements at wave 4.

**eTable 6.** Hazard ratios and 95% confidence intervals of incident frailty by global physical activity trajectories, further excluding participants reported daily activities difficulties during waves 1 to 4.

| **Global physical activity trajectories** | **Events/Total** | **Risk for incident frailty^a^** | |
| --- | --- | --- | --- |
|  |  | **HR (95% CI)** | **P value** |
| Persistently low | 418/1741 | Reference | |
| Initially low then improving | 163/1613 | 0.62 (0.50, 0.76) | <0.001 |
| Initially high then declining | 50/160 | 0.80 (0.59, 1.10) | 0.168 |
| Persistently moderate | 405/1778 | 0.66 (0.56, 0.77) | <0.001 |
| Persistently high | 224/1785 | 0.42 (0.35, 0.50) | <0.001 |

^a^ Adjusted for age, sex, ethnicity, education, cohabitation status, current smoking, alcohol consumption, depressive symptoms, hypertension, diabetes, stroke, cardiovascular diseases, chronic lung diseases, cancer, and BMI.

**eTable 7.** Non-response analysis comparing baseline characteristics between included and excluded participants of the ELSA.

| **Characteristics ^a^** | **Included**  **N=8227** | **Excluded**  **N=2823** | **P value ^b^** |
| --- | --- | --- | --- |
| Male (%) | 3780 (45.9%) | 1145 (40.6%) | <0.001 |
| Age (years) | 57.8±9.2 | 63.1±11.5 | <0.001 |
| White (%) | 7945 (96.6%) | 2702 (95.7%) | 0.041 |
| High level education (%) | 2810 (34.2%) | 541 (19.2%) | <0.001 |
| Living alone (%) | 1400 (17.0%) | 804 (28.5%) | <0.001 |
| Current smoking (%) | 757 (9.2%) | 391 (13.9%) | <0.001 |
| Drinking ≥ once per week (%) | 3353 (40.8%) | 905 (32.1%) | <0.001 |
| Depressive symptoms (%) | 496 (6.0%) | 490 (17.4%) | <0.001 |
| Hypertension (%) | 2545 (30.9%) | 1235 (43.7%) | <0.001 |
| Diabetes (%) | 209 (2.5%) | 203 (7.2%) | <0.001 |
| Stroke | 91 (1.1%) | 124 (4.4%) | <0.001 |
| Cardiovascular disease (%) | 353 (4.3%) | 330 (11.7%) | <0.001 |
| Chronic lung disease (%) | 168 (2.0%) | 183 (6.5%) | <0.001 |
| Cancer (%) | 239 (2.9%) | 123 (4.4%) | <0.001 |
| BMI (kg/m^2^) | 27.7±4.8 | 29.2±5.9 | <0.001 |
| Frailty index | 0.07 (0.04-0.13) | 0.30 (0.17-0.43) | <0.001 |
| Timed 5 chair rises (s) | 10.9±3.6 | 13.8±5.7 | <0.001 |
| Hand grip strength (kg) | 33.0±11.2 | 26.5±11.7 | <0.001 |
| Gait speed (cm/s) | 98.4±28.0 | 69.5±29.3 | <0.001 |

^a^ Data are presented as mean ± SD, n (%), or median (quartile 1– quartile 3).

^b^ P value reported for differences between 2 groups using t-test, chi-square test, or Wilcoxon rank test.

**eTable 8.** Mean differences of changes in physical function between global physical activity trajectories among men.

| **Global physical activity trajectories** | **Hand grip strength (kg)** | |  | **Timed 5 chair rises (s)** | |  | **Gait speed (cm/s)** | |  |
| --- | --- | --- | --- | --- | --- | --- | --- | --- | --- |
|  | **β (95% Cl)** **^a^** | **P value** |  | **β (95% Cl)** **^a^** | **P value** |  | **β (95% Cl)** **^a^** | **P value** |  |
| Persistently low | Reference | |  | Reference | |  | Reference | |  |
| Initially low then improving | 1.868 (0.474, 3.262) | 0.009 |  | -0.532 (-0.915, -0.149) | 0.006 |  | 5.123 (0.141, 10.105) | 0.044 |  |
| Initially high then declining | 1.323 (-1.665, 4.311) | 0.385 |  | -0.926 (-1.928, 0.075) | 0.070 |  | 6.355 (-3.655, 16.364) | 0.213 |  |
| Persistently moderate | 1.169 (-0.112, 2.451) | 0.074 |  | -0.218 (-0.662, 0.225) | 0.334 |  | 5.924 (1.112, 10.737) | 0.016 |  |
| Persistently high | 1.590 (0.315, 2.865) | 0.015 |  | -0.769 (-1.201, -0.336) | <0.001 |  | 6.902 (2.030, 11.774) | 0.005 |  |

^a^ Adjusted for age, sex, ethnicity, education, cohabitation status, current smoking, alcohol consumption, depressive symptoms, hypertension, diabetes, stroke, cardiovascular diseases, chronic lung diseases, cancer, functional limitations, BMI, and physical function measurements at wave 4.

**eTable 9**. Mean differences of changes in physical function between global physical activity trajectories among women.

| **Global physical activity trajectories** | **Hand grip strength (kg)** | |  | **Timed 5 chair rises (s)** | |  | **Gait speed (cm/s)** | |  |
| --- | --- | --- | --- | --- | --- | --- | --- | --- | --- |
|  | **β (95% Cl)** **^a^** | **P value** |  | **β (95% Cl)** **^a^** | **P value** |  | **β (95% Cl)** **^a^** | **P value** |  |
| Persistently low | Reference | |  | Reference | |  | Reference | |  |
| Initially low then improving | 1.793 (0.936, 2.650) | <0.001 |  | -0.500 (-0.896, -0.104) | 0.013 |  | 4.641 (-0.401, 9.682) | 0.071 |  |
| Initially high then declining | 0.420 (-1.028, 1.869) | 0.570 |  | 0.478 (-0.394, 1.349) | 0.283 |  | 1.220 (-8.214, 10.653) | 0.800 |  |
| Persistently moderate | 0.868 (0.161, 1.575) | 0.016 |  | -0.733 (-1.127, -0.339) | <0.001 |  | 4.831 (0.955, 8.708) | 0.015 |  |
| Persistently high | 1.242 (0.518, 1.967) | <0.001 |  | -0.995 (-1.404, -0.585) | <0.001 |  | 10.074 (5.986, 14.161) | <0.001 |  |

^a^ Adjusted for age, sex, ethnicity, education, cohabitation status, current smoking, alcohol consumption, depressive symptoms, hypertension, diabetes, stroke, cardiovascular diseases, chronic lung diseases, cancer, functional limitations, BMI, and physical function measurements at wave 4.

**eTable 10.** Hazard ratios and 95% confidence intervals of incident frailty by global physical activity trajectories among men.

| **Global physical activity trajectories** | **Events/Total** | **Risk for incident frailty^a^** | |
| --- | --- | --- | --- |
|  |  | **HR (95% CI)** | **P value** |
| Persistently low | 233/872 | Reference | |
| Initially low then improving | 97/872 | 0.58 (0.44, 0.76) | <0.001 |
| Initially high then declining | 35/90 | 0.91 (0.62, 1.33) | 0.619 |
| Persistently moderate | 266/949 | 0.71 (0.58, 0.87) | 0.001 |
| Persistently high | 151/997 | 0.44 (0.35, 0.56) | <0.001 |

^a^ Adjusted for age, sex, ethnicity, education, cohabitation status, current smoking, alcohol consumption, depressive symptoms, hypertension, diabetes, stroke, cardiovascular diseases, chronic lung diseases, cancer, functional limitations, and BMI.

**eTable 11.** Hazard ratios and 95% confidence intervals of incident frailty by global physical activity trajectories among women.

| **Global physical activity trajectories** | **Events/Total** | **Risk for incident frailty^a^** | |
| --- | --- | --- | --- |
|  |  | **HR (95% CI)** | **P value** |
| Persistently low | 363/1167 | Reference | |
| Initially low then improving | 105/839 | 0.62 (0.48, 0.79) | <0.001 |
| Initially high then declining | 54/126 | 0.95 (0.70, 1.28) | 0.744 |
| Persistently moderate | 402/1305 | 0.70 (0.60, 0.83) | <0.001 |
| Persistently high | 160/1010 | 0.40 (0.32, 0.49) | <0.001 |

^a^ Adjusted for age, sex, ethnicity, education, cohabitation status, current smoking, alcohol consumption, depressive symptoms, hypertension, diabetes, stroke, cardiovascular diseases, chronic lung diseases, cancer, functional limitations, and BMI.

**eTable 12.** Mean differences of changes in physical function between global physical activity trajectories among people aged < 60 years.

| **Global physical activity trajectories** | **Hand grip strength (kg)** | |  | **Timed 5 chair rises (s)** | |  | **Gait speed (cm/s)** | |  |
| --- | --- | --- | --- | --- | --- | --- | --- | --- | --- |
|  | **β (95% Cl)** **^a^** | **P value** |  | **β (95% Cl)** **^a^** | **P value** |  | **β (95% Cl)** **^a^** | **P value** |  |
| Persistently low | Reference | |  | Reference | |  | Reference | |  |
| Initially low then improving | 2.078 (1.226, 2.929) | <0.001 |  | -0.428 (-0.707, -0.150) | 0.003 |  | 4.431 (-0.612, 9.475) | 0.085 |  |
| Initially high then declining | 1.823 (-0.490, 4.136) | 0.122 |  | -0.589 (-1.455, 0.277) | 0.182 |  | 10.539 (-0.090, 21.168) | 0.052 |  |
| Persistently moderate | 1.610 (0.676, 2.544) | 0.001 |  | -0.513 (-0.864, -0.162) | 0.004 |  | 4.688 (0.111, 9.264) | 0.045 |  |
| Persistently high | 1.518 (0.579, 2.458) | 0.002 |  | -0.600 (-0.950, -0.249) | <0.001 |  | 10.609 (5.875, 15.342) | <0.001 |  |

^a^ Adjusted for age, sex, ethnicity, education, cohabitation status, current smoking, alcohol consumption, depressive symptoms, hypertension, diabetes, stroke, cardiovascular diseases, chronic lung diseases, cancer, functional limitations, BMI, and physical function measurements at wave 4.

**eTable 13**. Mean differences of changes in physical function between global physical activity trajectories among people aged ≥ 60 years.

| **Global physical activity trajectories** | **Hand grip strength (kg)** | |  | **Timed 5 chair rises (s)** | |  | **Gait speed (cm/s)** | |  |
| --- | --- | --- | --- | --- | --- | --- | --- | --- | --- |
|  | **β (95% Cl)** **^a^** | **P value** |  | **β (95% Cl)** **^a^** | **P value** |  | **β (95% Cl)** **^a^** | **P value** |  |
| Persistently low | Reference | |  | Reference | |  | Reference | |  |
| Initially low then improving | 0.900 (-1.390, 3.189) | 0.441 |  | -1.061 (-1.715, -0.408) | 0.001 |  | 5.567 (0.628, 10.506) | 0.027 |  |
| Initially high then declining | -0.737 (-2.723, 1.248) | 0.467 |  | 0.424 (-0.605, 1.452) | 0.419 |  | -0.703 (-9.561, 8.155) | 0.876 |  |
| Persistently moderate | -0.568 (-1.777, 0.641) | 0.357 |  | -0.356 (-0.875, 0.163) | 0.178 |  | 5.526 (1.571, 9.480) | 0.006 |  |
| Persistently high | 0.233 (-1.019, 1.484) | 0.716 |  | -1.074 (-1.608, -0.539) | <0.001 |  | 6.207 (2.109, 10.305) | 0.003 |  |

^a^ Adjusted for age, sex, ethnicity, education, cohabitation status, current smoking, alcohol consumption, depressive symptoms, hypertension, diabetes, stroke, cardiovascular diseases, chronic lung diseases, cancer, functional limitations, BMI, and physical function measurements at wave 4.

**eTable 14.** Hazard ratios and 95% confidence intervals of incident frailty by global physical activity trajectories among people aged < 60 years.

| **Global physical activity trajectories** | **Events/Total** | **Risk for incident frailty^a^** | |
| --- | --- | --- | --- |
|  |  | **HR (95% CI)** | **P value** |
| Persistently low | 288/1349 | Reference | |
| Initially low then improving | 126/1401 | 0.53 (0.42, 0.68) | <0.001 |
| Initially high then declining | 21/81 | 0.79 (0.48, 1.28) | 0.335 |
| Persistently moderate | 212/1092 | 0.59 (0.48, 0.74) | <0.001 |
| Persistently high | 105/1149 | 0.34 (0.26, 0.44) | <0.001 |

^a^ Adjusted for age, sex, ethnicity, education, cohabitation status, current smoking, alcohol consumption, depressive symptoms, hypertension, diabetes, stroke, cardiovascular diseases, chronic lung diseases, cancer, functional limitations, and BMI.

**eTable 15.** Hazard ratios and 95% confidence intervals of incident frailty by global physical activity trajectories among people aged ≥ 60 years.

| **Global physical activity trajectories** | **Events/Total** | **Risk for incident frailty^a^** | |
| --- | --- | --- | --- |
|  |  | **HR (95% CI)** | **P value** |
| Persistently low | 308/690 | Reference | |
| Initially low then improving | 76/310 | 0.68 (0.52, 0.90) | 0.006 |
| Initially high then declining | 68/135 | 0.99 (0.75, 1.30) | 0.925 |
| Persistently moderate | 456/1162 | 0.74 (0.63, 0.87) | <0.001 |
| Persistently high | 206/858 | 0.46 (0.38, 0.56) | <0.001 |

^a^ Adjusted for age, sex, ethnicity, education, cohabitation status, current smoking, alcohol consumption, depressive symptoms, hypertension, diabetes, stroke, cardiovascular diseases, chronic lung diseases, cancer, functional limitations, and BMI.


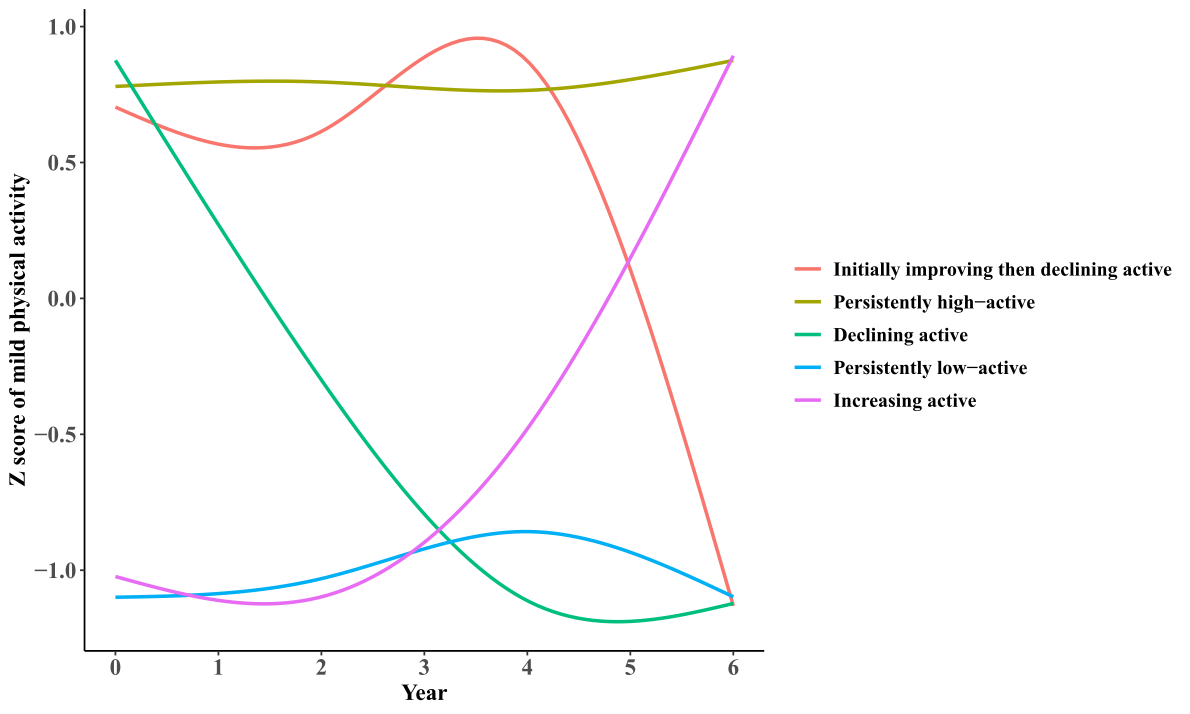


**eFigure 1.** Trajectories of participation in mild intensity physical activities by participants from the ELSA over a 6-year span.

Identified trajectories included: 1) persistently low (N=319); 2) initially low then improving (N=3027); 3) initially high then declining (N=96); 4) persistently high (N=4600); 5) initially improving then declining (N=185).


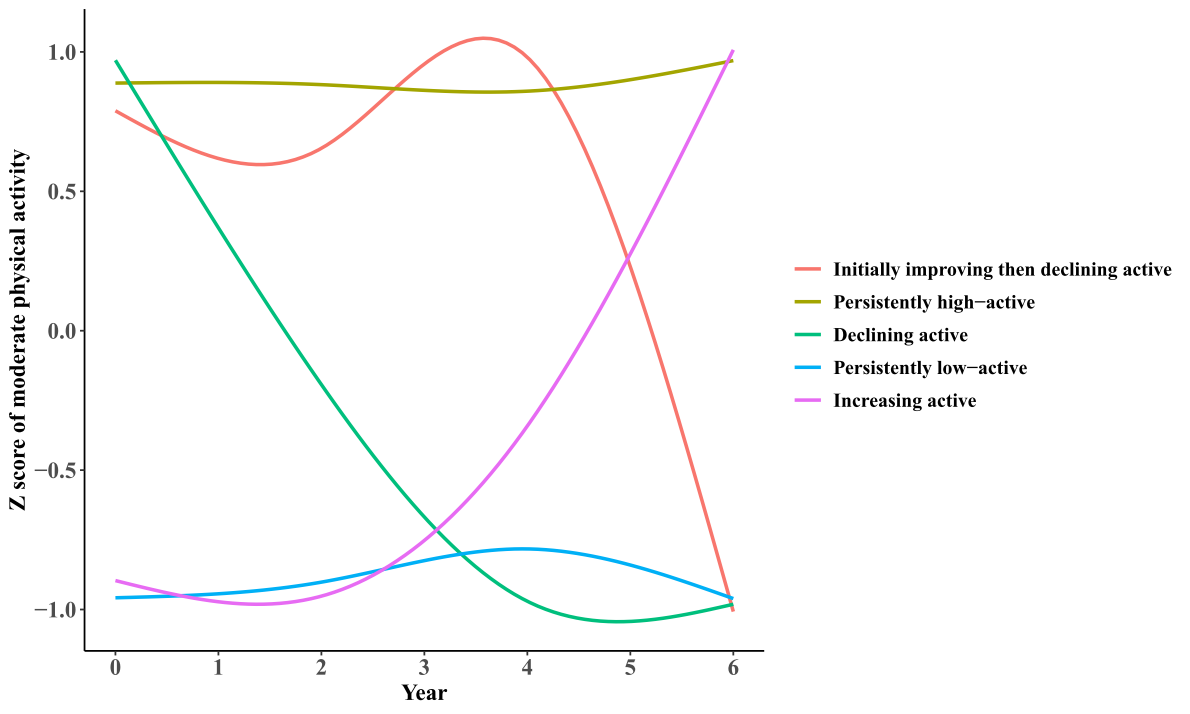


**eFigure 2.** Trajectories of participation in moderate intensity physical activities by participants from the ELSA over a 6-year span.

Identified trajectories included: 1) persistently low (N=545); 2) initially low then improving (N=2963); 3) initially high then declining (N=195); 4) persistently high (N=4252); 5) initially improving then declining (N=272).


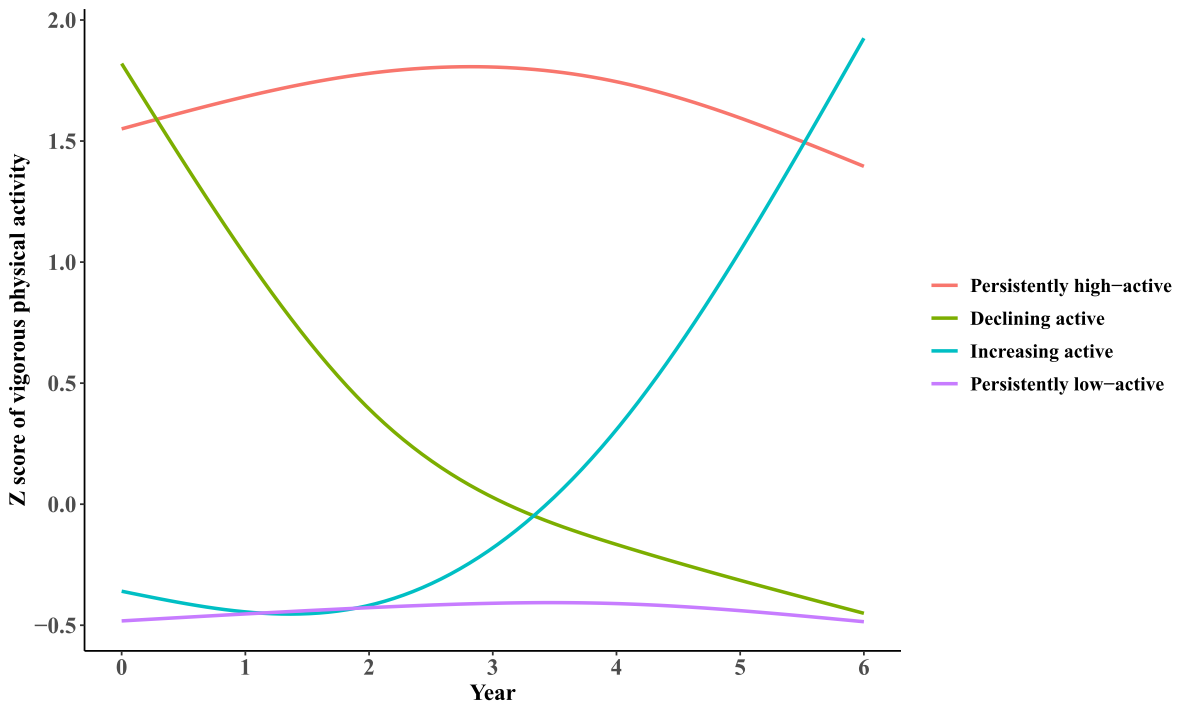


**eFigure 3.** Trajectories of participation in vigorous intensity physical activities by participants from the ELSA over a 6-year span.

Identified trajectories included: 1) persistently low (N=4049); 2) initially low then improving (N=1845); 3) initially high then declining (N=791); 4) persistently high (N=1542).
